# Diagnostic performance of the Bosniak classification, version 2019 for cystic renal masses: A systematic review and meta-analysis

**DOI:** 10.3389/fonc.2022.931592

**Published:** 2022-10-18

**Authors:** Qing Zhang, Xiaoli Dai, Wei Li

**Affiliations:** Department of Medical Imaging, Jiangsu Vocational College of Medicine, Yancheng, China

**Keywords:** Bosniak classification, mri, ct, cystic renal masses, systematic review

## Abstract

**Purpose:**

To systematically assess the diagnostic performance of the Bosniak classification, version 2019 for risk stratification of cystic renal masses.

**Methods:**

We conducted an electronic literature search on Web of Science, MEDLINE (Ovid and PubMed), Cochrane Library, EMBASE, and Google Scholar to identify relevant articles between June 1, 2019 and March 31, 2022 that used the Bosniak classification, version 2019 for risk stratification of cystic renal masses. Summary estimates of sensitivity, specificity, positive likelihood ratio (LR+), negative likelihood ratio (LR−), and diagnostic odds ratio (DOR) were pooled with the bivariate model and hierarchical summary receiver operating characteristic (HSROC) model. The quality of the included studies was assessed with the Quality Assessment of Diagnostic Accuracy Studies-2 tool.

**Results:**

A total of eight studies comprising 720 patients were included. The pooled sensitivity and specificity were 0.85 (95% CI 0.79–0.90) and 0.68 (95% CI 0.58–0.76), respectively, for the class III/IV threshold, with a calculated area under the HSROC curve of 0.84 (95% CI 0.81–0.87). The pooled LR+, LR−, and DOR were 2.62 (95% CI 2.0–3.44), 0.22 (95% CI 0.16–0.32), and 11.7 (95% CI 6.8–20.0), respectively. The Higgins *I* ^2^ statistics demonstrated substantial heterogeneity across studies, with an *I* ^2^ of 57.8% for sensitivity and an *I* ^2^ of 74.6% for specificity. In subgroup analyses, the pooled sensitivity and specificity for CT were 0.86 and 0.71, respectively, and those for MRI were 0.87 and 0.67, respectively. In five studies providing a head-to-head comparison between the two versions of the Bosniak classification, the 2019 version demonstrated significantly higher specificity (0.62 *vs.* 0.41, *p* < 0.001); however, it came at the cost of a significant decrease in sensitivity (0.88 *vs.* 0.94, *p* = 0.001).

**Conclusions:**

The Bosniak classification, version 2019 demonstrated moderate sensitivity and specificity, and there was no difference in diagnostic accuracy between CT and MRI. Compared to version 2005, the Bosniak classification, version 2019 has the potential to significantly reduce overtreatment, but at the cost of a substantial decline in sensitivity.

## Introduction

Over the past few decades, the incidence of renal cell carcinoma (RCC) has steadily increased in the United States and worldwide, with cross-sectional imaging playing an important role in diagnosis (1, 2). Indeed, as many as 70% of RCCs are detected incidentally in imaging studies for unrelated medical conditions (3). Since the Bosniak classification was initially proposed in 1986 (4), it has been widely used in clinical practice for risk stratification of cystic renal masses (5–7). According to the Bosniak classification, renal masses are stratified into categories (I, II, IIF, III, and IV) on the basis of CT imaging features. In general, Bosniak class I–II masses are “clearly benign” and do not require follow-up; Bosniak class IIF masses warrant follow-up by imaging; Bosniak III masses are considered indeterminate and are generally resected; and Bosniak IV masses are considered malignant and require surgical resection (8). Nevertheless, the American Urological Association considers active surveillance an option for Bosniak class III or IV lesions that are smaller than 2 cm if the patient is elderly, has limited life expectancy, or has other factors that preclude surgery. Previous studies have reported that approximately 50% of Bosniak III masses and 90% of Bosniak IV masses are malignant; however, the true prevalence of malignancy in Bosniak IIF masses is unknown (9). The natural history of Bosniak IIF–IV cysts is difficult to predict, and prior studies have cited risk factors including lesion size, body mass index, and previous history of renal cell carcinoma as relevant for decision-making. 

Although the Bosniak classification has long been preferred by radiologists and urologists for the management of cystic renal masses, the original version has its shortcomings. A previous systematic review demonstrated that the inter-reader agreement for Bosniak categorizations varied widely, ranging from 6% to 75% (6). Moreover, other studies have reported that nearly half of class III masses categorized according to the Bosniak classification are benign; however, these lesions are often recommended for surgical resection (5, 6, 10, 11). Therefore, a revised Bosniak classification was released in 2019, intended to improve inter-reader agreement through clearer definitions of the descriptors and to improve the specificity for predicting malignancy (9). In the Bosniak classification, version 2019, previously subjective descriptions such as thin (vs. thick) walls, few (vs. many) septa, and nodule (vs. an irregularly thickened wall or septa) have been explicitly defined: e.g., “thin” is now defined as ≤2 mm, “minimally thick” as 3 mm, and “thick” as ≥4 mm; “few” is defined as 1–3 septa and “many” as ≥4 septa. The designation of a cystic mass requires no more than 25% enhancement to differentiate these lesions from a potentially more aggressive neoplasm with necrosis. In particular, lesion size is excluded from the criterion.

Another major revision in the Bosniak classification, version 2019 is the formal incorporation of MRI criteria, which can provide more details about the walls or septa of cystic renal masses (12, 13). Notably, it has been reported that inter-reader agreement among experienced readers is higher with MRI (14).

Since the Bosniak classification, version 2019 was released, several studies evaluating the diagnostic accuracy of the new version have been published, including those that incorporate CT and MRI, either alone or in combination. Nevertheless, the diagnostic accuracy of the new version has not been systematically assessed. Thus, in this meta-analysis, we aimed to evaluate the performance of the Bosniak classification, version 2019 for risk stratification of cystic renal masses.

## Methods

This meta-analysis and systematic review was conducted in compliance with the Preferred Reporting Items for Systematic Reviews and Meta-Analyses (PRISMA) statement and performed with a predefined review and data extraction protocol (15). The primary outcome of our study was the diagnostic accuracy of the Bosniak classification, version 2019 for differentiating benign and malignant cystic renal masses. In addition, we aimed to compare the performance of the Bosniak classification, version 2019 with version 2005 in studies providing a head-to-head comparison.

### Search strategy and selection criteria

A thorough systematic literature search was performed on PubMed, EMBASE, Cochrane Library, Web of Science, and Google Scholar to identify peer-reviewed articles between 2019 and 31 March 2022. Results were sorted using Medical Subject Headings (MeSH) and the language was restricted to English. The search terms used are as follows: ([kidney] OR (renal) OR (nephron)] AND [(cancer) OR (mass*) OR (lesion*)]. An additional search was performed by manually screening the bibliographies of the included reviews and articles. Studies identified by the literature search were assessed by two independent reviewers (Z.Q., with 6 years of experience in performing systematic reviews and meta-analyses, and D.X.L., with 8 years of experience), and disagreements were resolved by consensus *via* discussion with a third reviewer (L.W.).

### Inclusion and exclusion criteria

We included studies that satisfied all of the following criteria: 1) use of CT or MRI as recommended in the Bosniak classification, version 2019; 2) reports the true positive (TP), false positive (FP), false negative (FN), true negative (TN), or other details for the reconstruction of 2 × 2 tables to evaluate the diagnostic performance; and 3) includes pathological results or at least 5 years’ follow-up as the reference standard.

We excluded studies that met any of the following criteria: 1) does not use the Bosniak classification, version 2019; 2) small sample size (fewer than 10 participants); 3) lack of sufficient detail for evaluating diagnostic performance; and 4) reviews, letters, guidelines, conference abstracts, or editorials.

### Data extraction and quality assessment

We used a predefined standardized form to extract relevant information, as follows: 1) demographic characteristics such as the number of patients and masses, patient age, male/female ratio, and tumor size; 2) study characteristics such as first author, publication year, location and period of the study conducted, number of readers and their experience, inter-reader agreement, blinding to final results, and reference standard; and 3) technical characteristics such as sequences and magnetic field strengths for MRI, or the number of slices and thickness for CT. We employed the Quality Assessment of Diagnostic Accuracy Studies–2 tool to evaluate the quality of studies and the likelihood of bias (16), in which the risk of bias for each study was assessed according to four domains: patient selection, method of the index test (parameter measurement and use of appropriate threshold to classify lesions), use of pathological results as the reference standard, and flow and timing.

### Data synthesis and statistical analysis

The bivariate model and hierarchical summary receiver operating characteristic (HSROC) model were used to estimate the sensitivity, specificity, positive likelihood ratio (LR+), negative likelihood ratio (LR−), and diagnostic odds ratio (DOR), along with their 95% confidence intervals (17, 18). In addition, we constructed forest plots and HSROC curves to graphically present the results. The publication bias of the included studies was estimated with Deeks’ funnel plot, and statistical significance was decided with Deeks’ asymmetry test, with a *p*-value less than 0.05 indicating publication bias (19).

Heterogeneity throughout the studies was determined with *Q* statistics and the inconsistency index (*I* ^2^), as follows: for values between 0% and 40%, unimportant; between 30% and 60%, moderate; between 50% and 90%, substantial; and between 75% and 100%, considerable (20). All analyses were performed with STATA 16.0 (StataCorp, Texas, USA), with *p*-values <0.05 considered statistically significant. Two reviewers (Z.Q. and D.X.L.) independently performed the data extraction and quality assessment, and disagreements were resolved through discussion and arbitrated by a third reviewer (L.W.).

We performed multiple subgroup analyses to investigate various clinical settings: 1) use of CT alone; 2) use of MRI alone; and 3) use of a combination of CT and MRI. Moreover, a pair of summary receiver operating characteristic (ROC) regions were drawn to compare the Bosniak classification, version 2019 with version 2005.

## Results

### Literature search and data extraction

**Figure 1:**
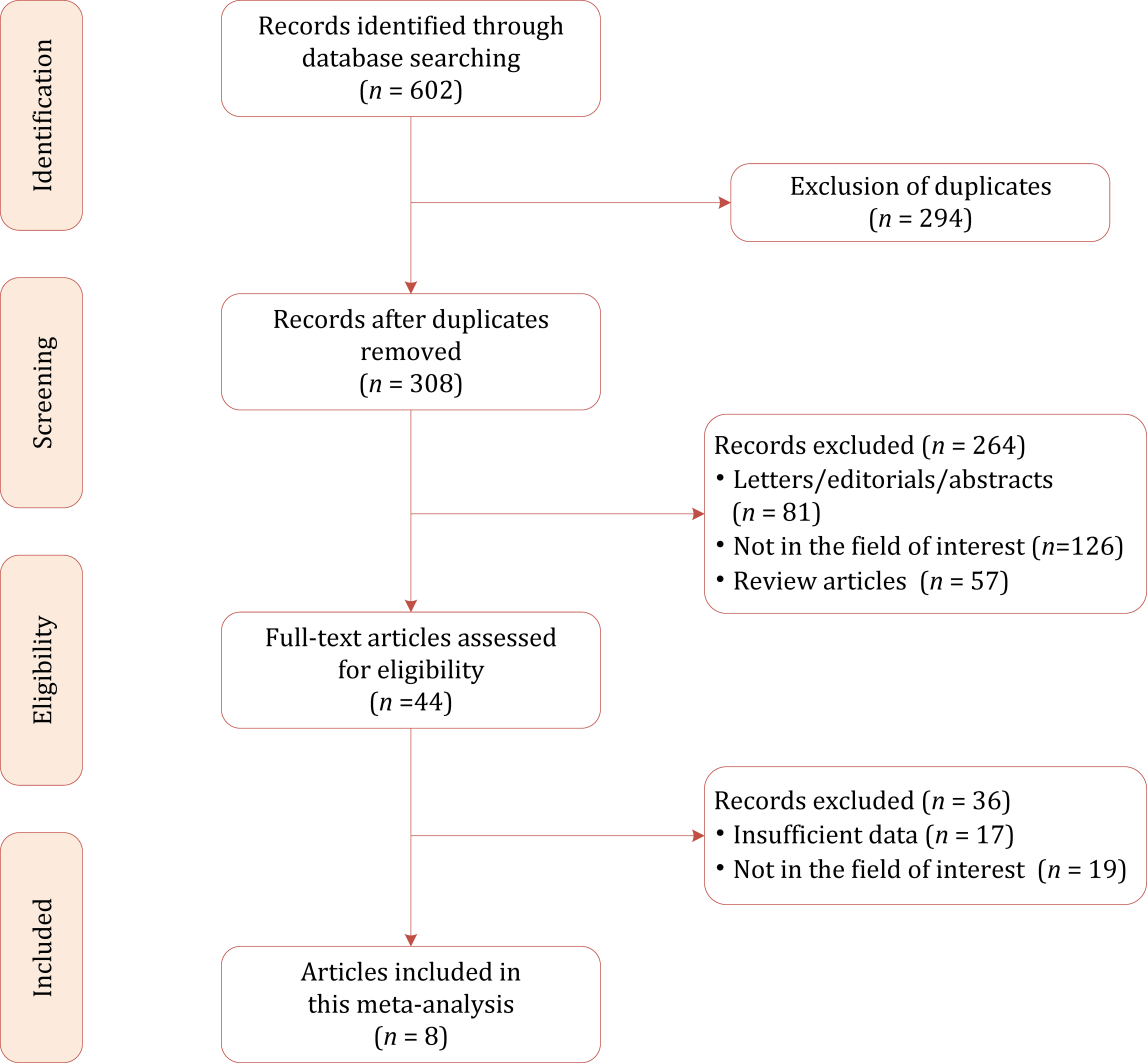
Study selection process for the systematic review and meta-analysis.

**Figure 1** shows the flowchart of the publication selection process. Our literature search initially identified 602 references, of which 294 were excluded as duplicates. After inspection of the titles and abstracts, a total of 264 articles were excluded. The full-text review was conducted among the remaining 44 potential articles, after which 36 articles were excluded for insufficient data or being outside the field of interest (e.g., in terms of inter-reader agreement, growth kinetics, or inclusion of class IIF/III/IV masses only). Ultimately, a total of eight articles comprising 720 patients were included in the current meta-analysis (21–28).

### Characteristics of the included studies

The detailed demographic and study characteristics are summarized in **Tables 1**, **2**. All studies included were retrospective in design, with sample sizes ranging from 50 to 207 patients and a mean participant age of 49 years–61.3 years. The average tumor size was between 25.4 mm and 65.9 mm, with a malignancy rate of 18.8%–78.1%. Regarding the imaging modalities, four studies reported results from CT (21, 23, 25, 28),, five reported results from MRI (22, 23, 25, 26, 28),, and three reported a combination of results from CT and MRI (21, 24, 27). With respect to the number of radiologists, only one study reported that the images were interpreted by only one reader (24), whereas in the other seven studies, the images were interpreted by at least two readers. The reported years of experience for image readers ranged from 1 to 33 years, with inter-reader agreements between 0.44 and 0.8 (measured with kappa value). In the majority of studies, the pathology results of resected tissue were used as the reference standard; however, in two studies, the availability of at least five years of follow-up was also used when pathology results were not available (21, 23).

**Table 1:** Demographic characteristics of the included studies.

Study	Country	Year	No. of patients	No. of masses	Malignant	Gender (M/F)	Age (year, mean ± SD)	Mass size (mm, mean ± SD)
Arita et al.	Japan	2022	83	103	28	56/27	59 (27–85)*	25.4 (9–160)*
Bai et al.	China	2020	207	207	96	139/68	49 ± 12	48.3 ± 20.7
Chan et al.	Canada	2021	65	65	25	43/22	63 ± 13	38.4 ± 26.8
Elbanna et al.	Canada	2021	79	80	15	51/28	61.3 ± 13.4	35 ± 21
Park et al.	Korea	2021	100	104	74	68/32	52.4 ± 11.6	35 ± 4
Tse et al.	USA	2020	50	59	38	56/44	55 ± 15	41 ± 34
Yan et al.	Canada	2021	73	73	73	45/28	60 ± 13	NA
Almalki et al.	Egypt	2022	63	67	41	34/29	49.5 ± 11.9	65.9 ± 21.0

NA, not available; SD, standard deviation.

^*^Median and interquartile range.

**Table 2:** Characteristics of the included studies.

Study	Design type	Period	No. of imaging readers	Experience (year/month)	Blinded	Examination	Parameters	*κ* value	Reference
**Arita et al.**	Retrospective	2010.08–2016.01	3	7/9/33	Yes	CT/CT + MRI	CT: 128 slices; MRI: 1.5 T; T1/T2/DWI/DCE	0.67/0.69	Pathological + 5 years follow-up
**Bai et al.**	Retrospective	2009.01–2019.06	8	1–21	Yes	MRI	MRI: 1.5–3.0 T; T1/T2/DCE	0.64	Pathological results
**Chan et al.**	Retrospective	2009–2019	3	1/1/2	Yes	CT/MRI	CT: 16–256 slices; MRI: 1.5 T–3.0 T; T1/T2/DWI	0.44/0.39	Pathological + 5 years follow-up
**Elbanna et al.**	Retrospective	2009–2018	1	5	Yes	CT + MRI	CT: 64 slices; MRI: 1.5 T; T1/T2/DCE	0.68	Pathological results
**Park et al.**	Retrospective	2010.01–2019.12	2	5/8	Yes	CT/MRI	CT: 16–64 slices; MRI: 1.5–3.0 T; T1/T2/DWI/DCE	0.80/0.76	Pathological results
**Tse et al.**	Retrospective	2005–2019	2	12/14	Yes	MRI	MRI: 1.5–3.0 T; T1/T2/DWI/DCE	0.55	Pathological results
**Yan et al.**	Retrospective	2009–2019	3	1/1/2	Yes	CT + MRI	CT: 16–256 slices; MRI: 1.5 T–3.0 T; T1/T2/DWI	0.80	Pathological results
**Almalki et al.**	Prospective	2019.08–2022.02	3	>10	Yes	CT/MRI	CT: 64–128 slices; MRI: 1.5 T; T1/T2/DWI/DCE	0.66/0.62	Pathological results

DCE, dynamic contrast–enhanced; DWI, diffusion-weighted imaging; T1, T1-weighted imaging; T2, T2-weighted imaging.

### Quality assessment

**Figure 2:**
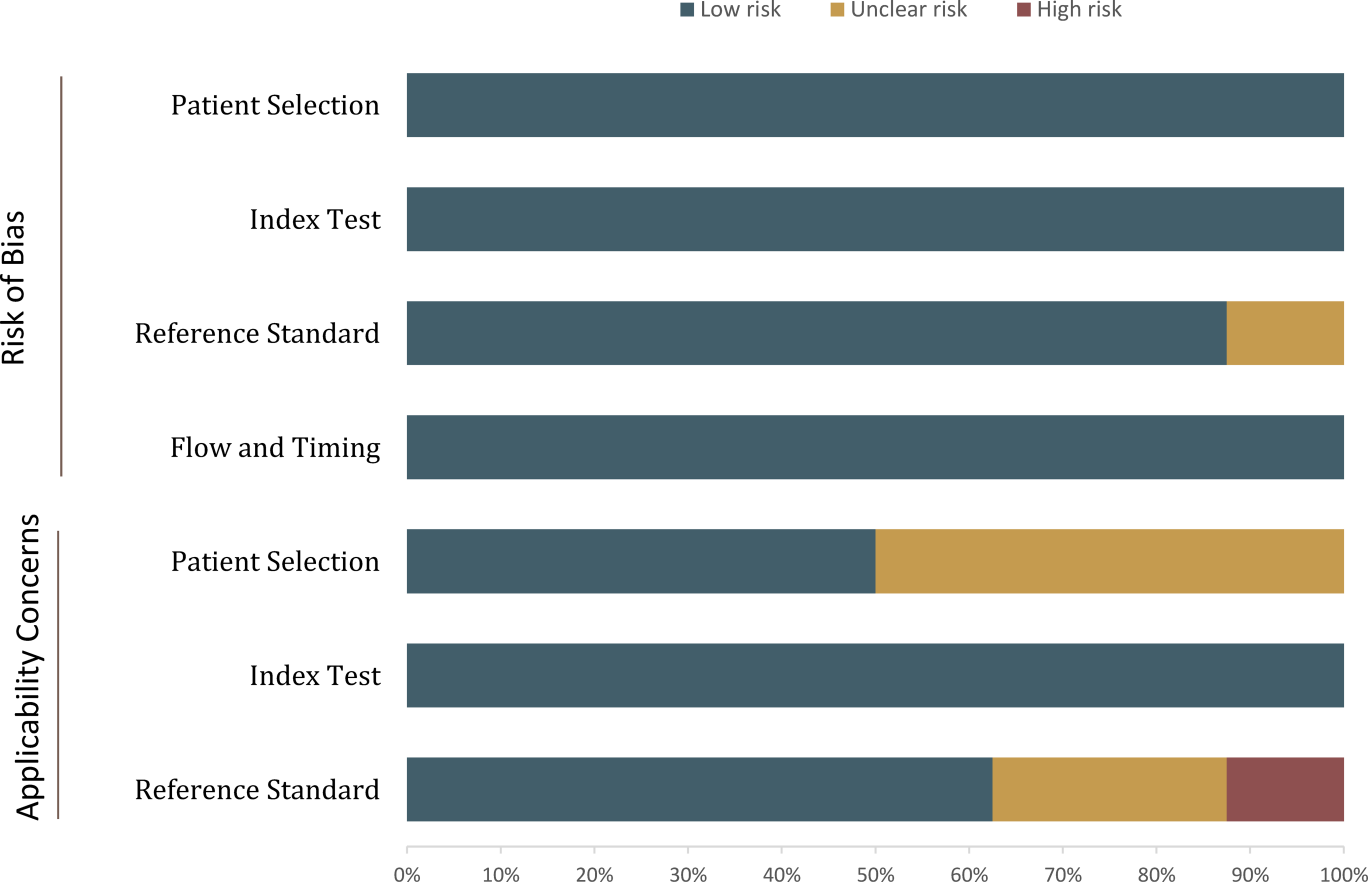
Grouped bar charts showing the risk of bias and concerns regarding the applicability of the included studies.

The overall quality of the included studies was not very high, primarily due to patient selection (**Figure 2**). In more than half of the studies, the analysis was restricted to cystic renal masses with confirmed pathological results (23, 25–28), which may have led to selection and verification biases, as those masses under surveillance that did not undergo histological confirmation were not included. Regarding the reference standard, two of the studies used the availability of at least five years of follow-up data, rather than histopathological findings, as the reference standard for some patients (21, 22). In terms of flow and timing, all included studies were assigned a low risk of bias.

### Diagnostic performance of the Bosniak classification, version 2019

**Figure 3:**
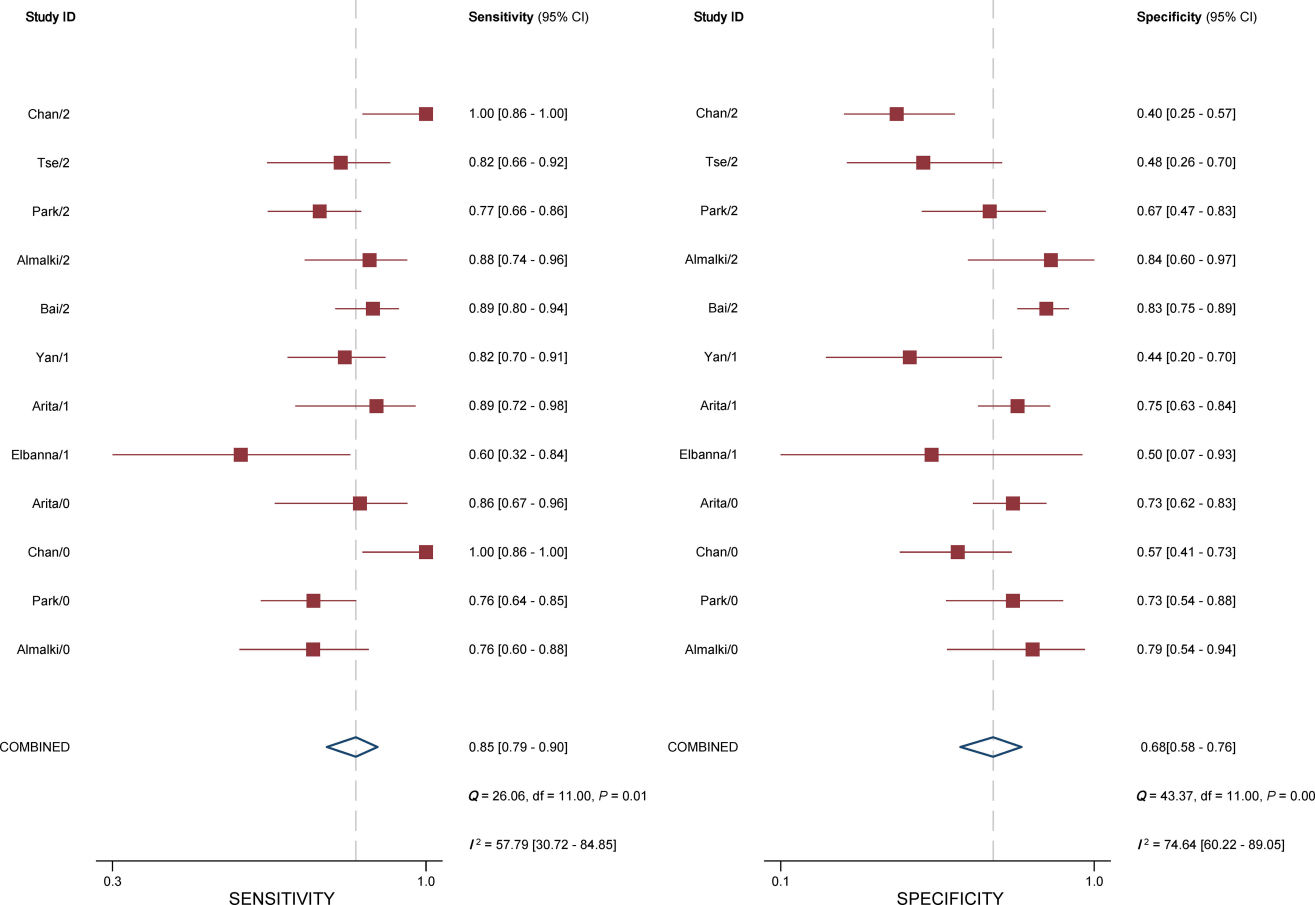
Coupled forest plot of pooled sensitivity and specificity. The numbers are pooled estimates; the 95% CI appears in parentheses. Corresponding heterogeneity statistics are provided in the bottom right corners. Horizontal lines indicate 95% confidence intervals.

**Figure 4:**
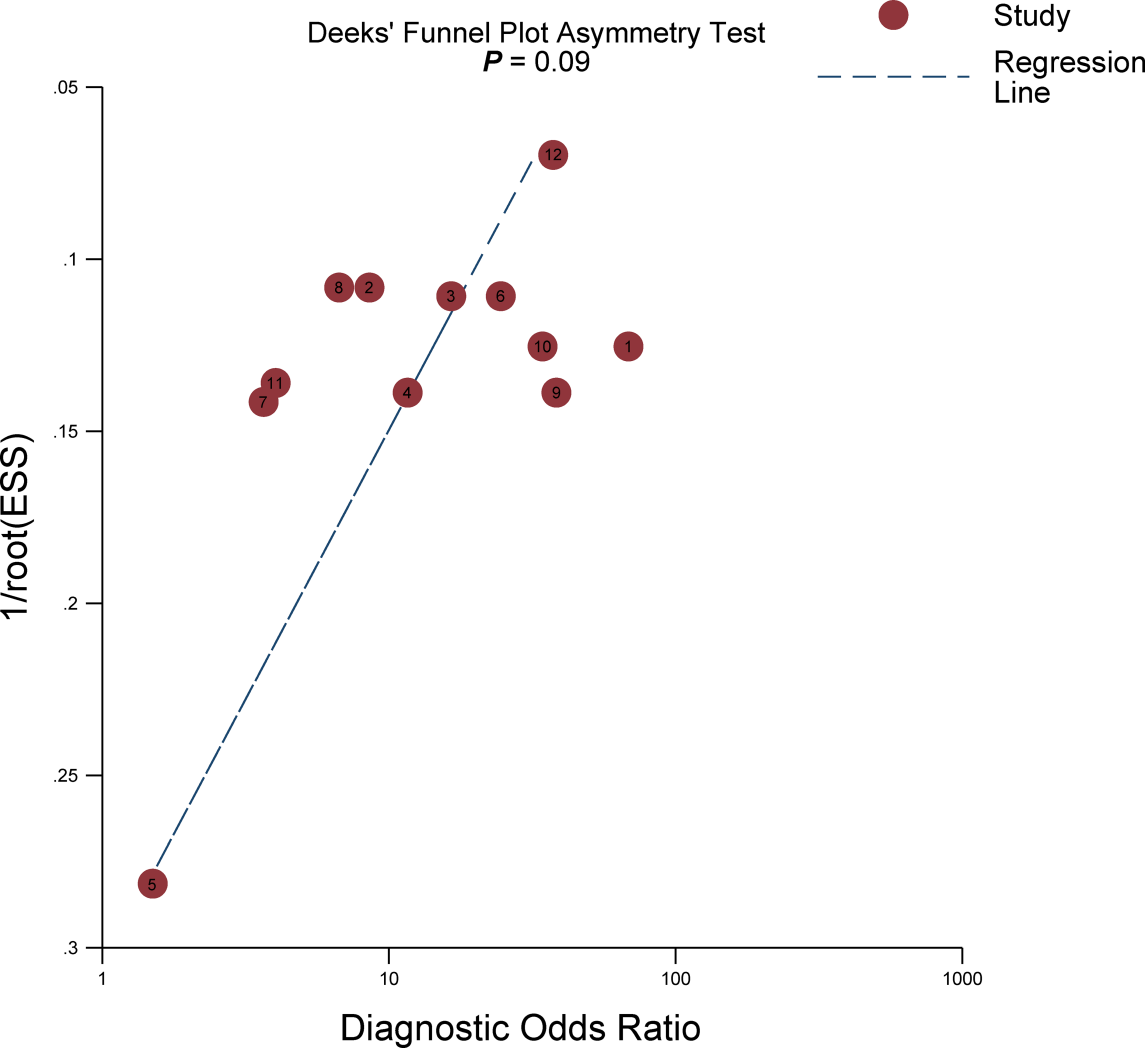
The Deeks’ funnel plot.

**Figure 5:**
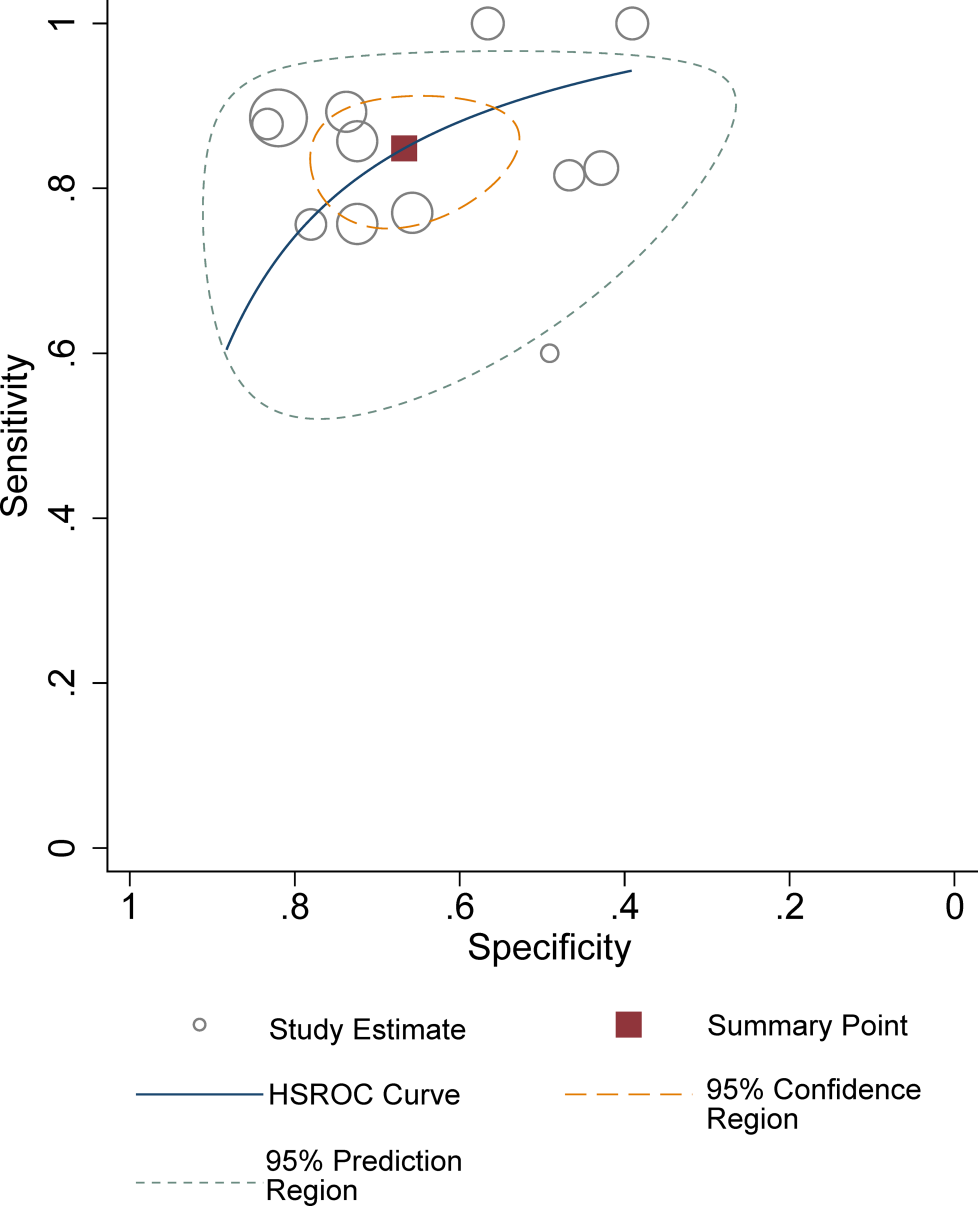
HSROC plots with summary point and 95% confidence area for the overall.

For individual studies, the sensitivity ranged from 0.76 to 1.00, with a specificity of 0.40–0.84. The summary estimates of sensitivity and specificity for all eight studies were 0.85 (95% CI 0.79–0.90) and 0.68 (95% CI 0.58–0.76), respectively; the corresponding coupled forest plots are presented in **Figure 3**. The pooled LR+, LR−, and DOR were 2.62 (95% CI 2.00–3.44), 0.22 (95% CI 0.16–0.32), and 11.7 (95% CI 6.8–20.0), respectively, with an area under the HSROC of 0.84 (95% CI 0.81–0.87). The *Q* test revealed substantial heterogeneity throughout the studies (*p* < 0.05), and the Higgins *I* ^2^ statistics indicated substantial heterogeneity in terms of both sensitivity (*I* ^2^ = 57.8%) and specificity (*I* ^2^ = 74.6%). Furthermore, a large difference between the 95% confidence region and the 95% prediction region in the HSROC curve suggested substantial heterogeneity (**Figure 4**). Deeks’ funnel plot is presented in **Figure 5**; the *p*-value of 0.09 for the slope coefficient indicates that the likelihood of publication bias was not statistically significant.

### Subgroup analyses

**Figure 6:**
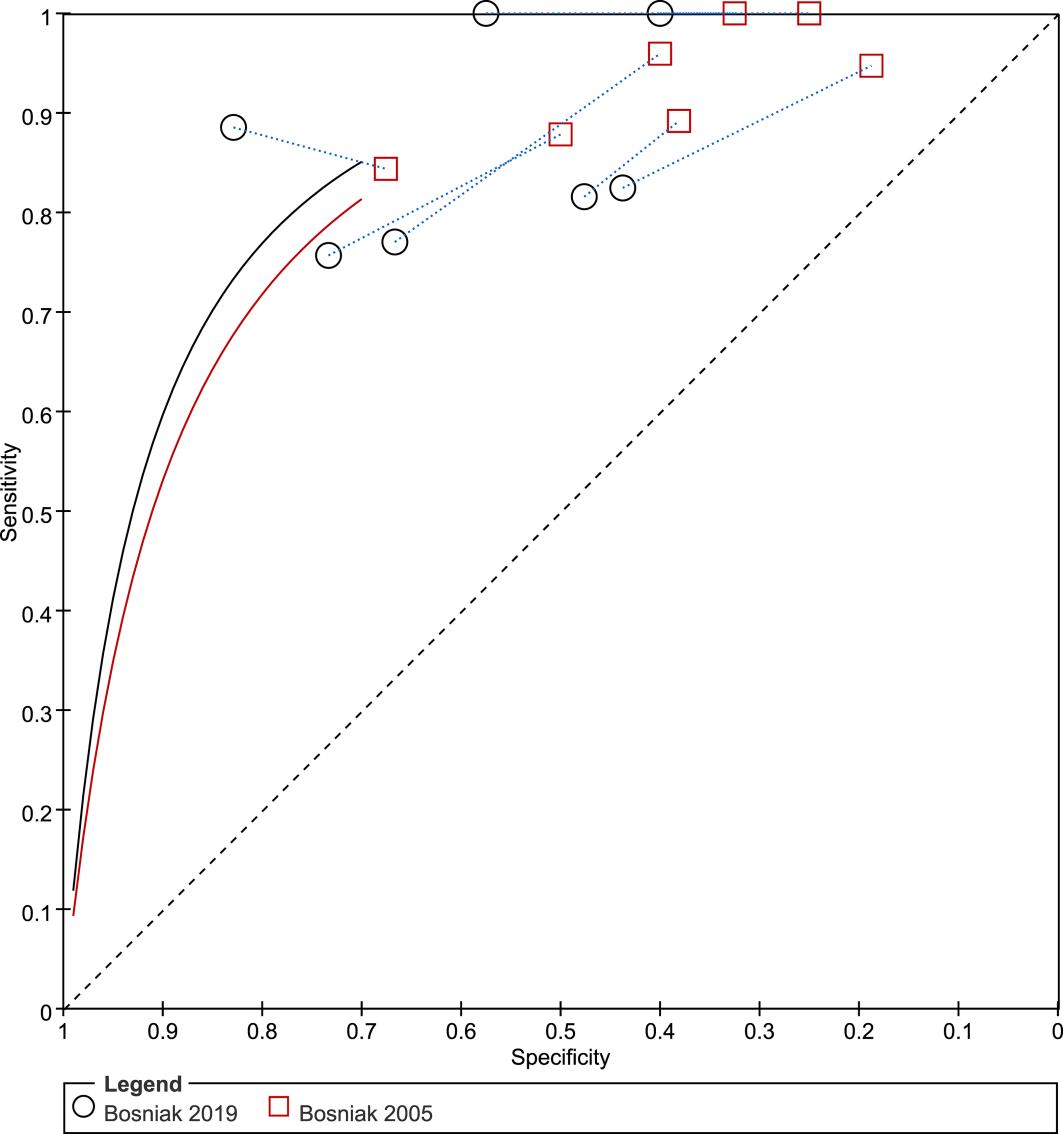
Summary receiver operating characteristic (ROC) performance curves for the Bosniak classification, version 2019 and version 2005.

We compared the Bosniak classification, version 2019 with version 2005 across five studies that provided a head-to-head comparison (22, 23, 25–27). The pooled sensitivity of 0.88 (95% CI 0.76–0.95) vs. 0.94 (95% CI 0.89–0.98), respectively, suggests that the new version is significantly inferior to the previous one (*p* = 0.001). However, the pooled specificity of 0.62 (95% CI 0.46–0.75) vs. 0.41 (95% CI 0.27–0.57), respectively, indicates that version 2019 could significantly reduce overtreatment and unnecessary resection (*p* < 0.001). In general, the comparable area under the ROC (both were 0.83, 95% CI 0.79–0.86) demonstrated no difference between the two Bosniak versions regarding overall diagnostic performance. A comparison of the summary ROC is presented in **Figure 6**.

We also performed subgroup analyses for various examinations involving CT and MRI, both alone and in combination. Regarding the four studies reporting the diagnostic accuracy of CT, the pooled sensitivity and specificity were 0.86 (95% CI 0.68–0.95) and 0.71 (95% CI 0.61–0.80), respectively (21, 23, 25, 28). For the five studies reporting the diagnostic accuracy of MRI, the pooled sensitivity was comparable (0.87, 95% CI 0.78–0.93) but the specificity was lower (0.67, 95% CI 0.48–0.81) (22, 23, 25, 26, 28). Regarding the three studies reporting the diagnostic accuracy of CT in combination with MRI, the pooled sensitivity (0.81, 95% CI 0.70–0.91) was lower than using CT or MRI alone, while the specificity was comparable (0.67, 95% CI 0.61–0.82) (21, 24, 27).

## Discussion

In this study, we systematically assessed the diagnostic performance of using the Bosniak classification, version 2019 for risk stratification of cystic renal masses. The pooled sensitivity and specificity, based on seven studies, demonstrated moderate diagnostic accuracy in differentiating benign and malignant lesions. In an earlier systematic review that had evaluated the previous Bosniak classification in use with CT, the pooled sensitivity and specificity, based on 35 studies, were 0.93 (95% CI 0.89–0.95) and 0.67 (95% CI 0.59–0.76), respectively; however, this offered only an indirect comparison (5). Given that a head-to-head comparison was reported in five of the included studies, we used them to compare the two Bosniak classification versions (22, 23, 25–27). According to our analyses, the Bosniak classification, version 2019 is significantly superior to version 2005 in terms of specificity (0.62 vs. 0.41, *p* < 0.001); however, it comes at the cost of a substantial decline in sensitivity (0.88 vs. 0.94, *p* = 0.001).

We noted significant heterogeneity among the included studies; however, because of the insufficient number of studies, it is unfeasible to perform meta-regression to investigate the sources of this heterogeneity. Nevertheless, we carried out subgroup analyses according to the examination methods used. The pooled sensitivity of 0.89 vs. 0.89 and pooled specificity of 0.69 vs. 0.63 indicated that the diagnostic accuracy between CT (21, 23, 25) and MRI (22, 23, 25, 26) was comparable, but that the combination of CT and MRI showed a trend of increasing specificity but declining sensitivity (21, 24, 27).

Currently, for patients with Bosniak IV masses, resection is recommended if there are no significant comorbidities; however, more and more evidence demonstrates that, for some Bosniak class III masses and even some Bosniak IV masses with small nodules, active surveillance of cystic renal masses is safe, especially for older patients with limited life expectancy and surgical comorbidities (29, 30). Nevertheless, Tse et al. found that class IV masses grow faster and are more likely to progress than class III masses, and therefore need more intensive surveillance (31).

In the Bosniak classification, version 2019, MRI is formally incorporated because it is particularly valuable for characterizing cystic renal masses that are indeterminate on CT. In the current meta-analysis, several studies providing a head-to-head comparison between MRI and CT reported that the inter-modality agreement was substantial, with a *κ* value of 0.63–0.78 (21, 25). Nevertheless, different modalities may lead to category redistribution (e.g., some cystic renal masses assigned by CT may be upgraded or downgraded by MRI) and result in management changes (12, 13). In the Bosniak classification, version 2019, any calcification is a feature of category II; however, thick or nodular calcification at CT may obscure enhancing features that could affect the classification. Thus, the Bosniak classification, version 2019 recommends that if thick or nodular calcification is identified at CT, the patient should be referred to MRI to determine the final classification. Nonetheless, this change did not significantly impact the overall classification assigned when comparing evaluation by CT or MRI; this was in line with our finding that CT and MRI have comparable diagnostic performance, even if CT showed a slightly higher specificity. Nevertheless, this result was derived from only a few studies; therefore, whether MRI is more likely to lead to category migration compared to CT needs to be validated in larger cohort studies.

A primary goal of the Bosniak classification, version 2019 was to improve inter-reader agreement between radiologists. In the present meta-analysis, all included studies reported the inter-reader agreement, with a kappa value of 0.44–0.80 for CT alone and 0.39–0.76 for MRI alone, demonstrating a moderate to substantial reproducibility among radiologists. However, the majority of the studies did not observe a substantial improvement in inter-reader agreement in the Bosniak classification, version 2019, suggesting that the updates to the classification system have not solved the previously identified issues with inter-reader variability. Moreover, one study found that the inter-reader agreement of the Bosniak classification, version 2019 was lower than that of version 2005, both for CT and MRI (32). Thus, efforts still need to be made to standardize imaging interpretations and improve inter-reader agreement in the future.

There are some limitations to our study. First, all studies included were retrospective in design, which carries a high risk of bias in terms of patient selection. However, it was unfeasible to pool the summary estimates from prospective studies because they are not available. Second, we noted substantial heterogeneity throughout the included studies, which affected the general applicability of our meta-analysis. Because of the small sample, it was impossible to perform meta-regression; however, we performed subgroup analyses with regard to different imaging methods. Third, our meta-analysis was based on only seven studies; thus, the results should be regarded with caution. Large prospective cohort studies are still needed to validate this new version of the Bosniak classification in clinical practice.

## Conclusion

The Bosniak classification, version 2019 has moderate sensitivity and specificity and no significant difference between CT and MRI in terms of diagnostic accuracy. As compared to the 2005 version, version 2019 has the potential to significantly reduce overtreatment, but at the cost of a substantial decline in sensitivity.

## Data availability statement

The original contributions presented in the study are included in the article/supplementary material. Further inquiries can be directed to the corresponding author.

## Author contributions

QZ and XD contributed equally to this work. All authors contributed to the article and approved the submitted version.

## Conflict of interest

The authors declare that the research was conducted in the absence of any commercial or financial relationships that could be construed as a potential conflict of interest.

## Publisher’s note

All claims expressed in this article are solely those of the authors and do not necessarily represent those of their affiliated organizations, or those of the publisher, the editors and the reviewers. Any product that may be evaluated in this article, or claim that may be made by its manufacturer, is not guaranteed or endorsed by the publisher.
